# Leveraging productivity indicators for anomaly detection in swine breeding herds with unsupervised learning

**DOI:** 10.3389/fvets.2025.1586438

**Published:** 2025-11-17

**Authors:** Mafalda Pedro Mil-Homens, Chong Wang, Giovani Trevisan, Fernanda Dórea, Daniel C. L. Linhares, Derald Holtkamp, Gustavo S. Silva

**Affiliations:** 1 Department of Veterinary Diagnostic and Production Animal Medicine, College of Veterinary Medicine, Iowa State University, Ames, IA, United States; 2 Department of Animal Health and Anatomy, Faculty of Veterinary Medicine, Autonomous University of Barcelona, Barcelona, Spain; 3 Department of Statistics, College of Liberal Arts and Sciences, Iowa State University, Ames, IA, United States; 4 Swedish Veterinary Agency, Uppsala, Sweden; 5 Food and Agriculture Organization of the United Nations, FAO, Rome, Lazio, Italy

**Keywords:** isolation forest, autoencoders, K-nearest neighbors, unsupervised machine learning, anomaly detection

## Abstract

**Introduction:**

In swine disease surveillance, obtaining labeled data for supervised learning models can be challenging because many farms lack standardized diagnostic routines and consistent health monitoring systems. Unsupervised learning is particularly suitable in such scenarios because it does not require labeled data, allowing for detecting anomalies without predefined labels. This study evaluates the effectiveness of unsupervised machine learning models in detecting anomalies in productivity indicators in swine breeding herds.

**Methods:**

Anomalies, defined as deviations from expected patterns, were identified in indicators such as abortions per 1000 sows, prenatal losses, preweaning mortality, total born, liveborn, culled sows per 1000 sows, and dead sows per 1000 sows. Three unsupervised models - Isolation Forest, Autoencoder, and K-Nearest Neighbors (KNN) - were applied to data from two swine production systems. The herd-week was used as the unit of analysis, and anomaly scores above the 75th percentile were used to flag anomalous weeks. A permutation test assessed differences between anomalous and non-anomalous weeks. Performance was evaluated using F1-score, precision, and recall, with true anomalous weeks defined as those coinciding with reported health challenges, including porcine reproductive and respiratory syndrome (PRRS) and Seneca Valley virus outbreaks. A total of 8,044 weeks were analyzed.

**Results:**

The models identified 336 anomalous weeks and 1,008 non-anomalous weeks in Production System 1, and 1,675 anomalous weeks and 5,025 non-anomalous weeks in Production System 2. The results from the permutation test revealed significant differences in productivity indicators between anomalous and non-anomalous weeks, especially during PRRS outbreaks, with more subtle changes observed during Seneca Valley virus outbreaks. The models performed well in detecting the PRRSV anomaly, achieving perfect precision (100%) across all models for both production systems. For anomalies like SVV the models showed lower performance compared to PRRSV.

**Discussion:**

These findings suggest that unsupervised machine learning models are promising tools for early disease detection in swine herds, as they can identify anomalies in productivity data that may signal health challenges.

## Introduction

The global pork production industry is crucial for food security, with the United States being one of the top producers and exporters (55). Maintaining herd health is essential for economic success in this sector. However, challenges persist, with diseases having a major impact on the swine production systems. Porcine reproductive and respiratory syndrome virus (PRRSV) was estimated to cost the U.S. swine industry around $1.2 billion annually (1). In the Netherlands, PRRSV outbreaks were estimated to cause an average loss of $131.2 per sow per outbreak (2). The introduction of the porcine epidemic diarrhea virus (PEDV) in the U.S. in 2013 caused the loss of seven million pigs (3), and a study made in the U.S. estimated the cost to be $300,000 per year for a single 700-sow farrow-to-finishing herd (4). In Europe the cost of classical swine fever outbreaks in the Netherlands and Belgium was estimated as $2.3 billion and $11.5 million, respectively. In Romania, African swine fever led to a reduction of the national pig population by 8.44%, with 348,691 pigs slaughtered (5), and a study estimated a loss of $15 billion in a scenario where African swine fever was introduced in the U.S. and controlled and estimated a loss of $50 billion in a scenario where the disease spread to feral swine and it was not controlled (6). These are some examples of the economic impact of diseases on production, and with that, it is important to consider strategies to detect early anomalies, trigger outbreak investigation, and implement measures to avoid the further spread of pathogens.

Anomalies are data points that deviate from values expected under a given model or contextual distribution. Such deviations may arise from measurement errors, atypical operating conditions, or previously unknown processes affecting the system (7). In the swine industry, an anomaly refers to an unexpected value in productivity indicators, which can arise for multiple reasons like animal management and husbandry, human error in data collection, environmental changes, and health-related factors. Numerous studies have demonstrated that syndromic surveillance is highly effective in detecting health-related events in swine breeding herds. For example, using statistical process control (SPC) charts to track productivity indicators has enabled the detection of deviations associated with PRRSV outbreaks early, often before routine diagnostic monitoring (8–10). These studies indicated that observing multiple productivity indicator patterns allows early detection of health challenges. Apart from these methods, anomaly detection also relies on methods such as time series (11, 12), supervised machine learning (13), and unsupervised learning (7, 14).

Unsupervised learning involves using algorithms to identify patterns or structures in datasets without any external guidance or labeled information (15). It can be used for different purposes, and while there is limited information on its use for disease surveillance in the swine industry, it has been utilized to test lying patterns in pigs by combining image processing, unsupervised clustering, and central neural networks with support vector machine classification (16). Unsupervised learning was also used to identify variables of economically important performance traits in swine (17) and to identify evolving phenotypes after trauma using principal component analysis (18). Moreover, unsupervised learning was used to describe the network structure and spatiotemporal characteristics of swine shipment using factor analysis for mixed data and hierarchical clustering (19). In the context of swine disease surveillance, obtaining labeled data for supervised learning models can be difficult because, in many cases, farms do not have established diagnostic routines or consistent health monitoring systems to generate reliable labels for anomalies, making it hard to train supervised models to detect these anomalies reliably. For this reason, unsupervised models are an attractive option since they do not require labeled data, allowing them to identify unknown anomalies that supervised learning might miss if not predefined in the labels (20–22). In this study, it was hypothesized that unsupervised models could be used to detect anomalies in production indicators (abortions per 1,000 sows, prenatal losses, preweaning mortality, total born, liveborn, culled sows per 1,000 sows and dead sows per 1,000 sows) commonly affected when there is disease introduction in swine breeding herds (23, 24) from farms that lack active surveillance or sufficient labeled data. These models could identify deviations from normal production patterns that might go unnoticed and could trigger further investigation of potential health challenges. This provides insight into how unsupervised learning might perform when applied to real-world settings, where labels are often unavailable, and emphasizes the importance of these models in filling the gap when traditional supervised learning approaches cannot be applied.

The primary goal of this study was to evaluate the efficacy of unsupervised machine learning models in detecting anomalies in the productivity indicators of swine breeding herds. Efficacy in this context was defined by the model's ability to identify anomalies indicative of health challenges based on deviations in productivity indicators. Specifically, the study aimed to (1) compare unsupervised models in terms of anomaly detection across anomalous and non-anomalous weeks and (2) examine the potential of unsupervised models to uncover hidden anomalies that could signal emerging health challenges, even in the absence of known disease labels.

## Methods

### Overview of the study

Data from two distinct production systems were used in the study, and the models were implemented for each system individually, allowing for a comprehensive evaluation across different settings, using the herd-week as the unit of analysis. Unsupervised learning offers benefits since it does not depend on labeled data, enabling the discovery of previously unidentified anomalies that supervised methods might overlook. In this study, three unsupervised models were implemented—Isolation Forest, Autoencoder, and K-nearest neighbors (KNN) to identify anomalous weeks (weeks that had anomaly scores above the 75th percentile) in productivity indicators without providing the models with prior knowledge of health challenges. Following model implementation, the identified anomalous weeks were compared with known health challenges to evaluate the potential of these models for syndromic surveillance in swine herds.

### Data source

The data used in this study originated from an ongoing surveillance system that monitors weekly productivity data from swine breeding herds from two swine production systems across the United States (FieldEPi Swine Data Surveillance System, https://fieldepi.org/ongoing-monitoring-of-production-data-breeding-sows/). This system collects weekly productivity data via automated emails or Application Programming Interface (API), securely stored on a dedicated server. This robust surveillance infrastructure and ongoing communication with participants enables the aggregation of sow performance monitoring data that contains productivity data on different indicators related to breeding, farrowing, and weaning performance, from various production systems, facilitating both retrospective and prospective studies.

### Inclusion criteria and dataset

The breeding herds selected for this analysis followed the inclusion criteria: (a) willingness to share weekly productivity indicators, (b) being operated at a continuous breeding-farrowing fashion (i.e., not batch farrowing), (c) willingness to share diagnostic data, vaccination data, and reports from health challenges from field veterinarians, (d) having at least 21 weeks of data, comprising a full production cycle in breeding herds.

The dataset was built with data from two production systems. Production System 1 had an inventory of 24,847 sows distributed in seven farms and 1,344 herd-weeks (i.e., weekly indicators reported per herd on a calendar-week basis) of productivity data. Production System 2 had an inventory of 205,257 sows, 42 farms, and 6,700 herd-weeks of productivity data. The indicators used as input for the analysis were the weekly abortions per 1,000 sows, weekly average number of total pigs born per litter, weekly average number of liveborn piglets per litter, weekly percentage of prenatal losses defined as the difference between total born and born alive per litter, weekly preweaning mortality percentage from pigs born alive, weekly females culled per 1,000 sows, and weekly dead sows per 1,000 sows.

Data on diagnostics provided by the production systems and reports of health challenges made by the veterinarians were also collected. The reported outbreaks comprised the following pathogens: Seneca Valley virus (SVV) and PRRSV. The impact of a disease on animals does not occur solely during the week of the outbreak. To reflect this more accurately, each disease's incubation and resolution periods were considered and added to the dataset as indicated in the literature. A period of 8 weeks before and 15 weeks after the week of a PRRSV outbreak positive diagnostic was considered as weeks within the outbreak period (10, 25). For SVV, the outbreak period was considered as the week before the positive diagnostic and 2 weeks after (25–27).

### Unsupervised learning for anomaly detection

The implementation and analysis using the three unsupervised models were done in the statistical programming environment R Statistical Software (v4.4.0; (56)) and Microsoft Excel.

### Isolation Forest

Isolation Forest operates by recursively partitioning the data using randomly selected features, isolating anomalous values faster than traditional methods such as clustering or distance-based techniques. An isolation tree is constructed by recursively partitioning the dataset based on randomly selected attributes and threshold values until reaching a specified height limit or a single data point (28, 29). This process generates a binary tree structure. To quantify anomalies, a ranking method evaluates each data point's path length from the root to the leaf node. The anomaly score [0, 1] is calculated using the formula:


$$\begin{array}{l} s\left(x,n\right)=2-E\left(h\left(x\right)\right)\times c\left(n\right), \end{array}$$

where E(h(x)) is the expected path length, *n* is the testing data size, and c(*n*) represents the average path length for unsuccessful searches in a binary search tree. Points with scores close to 1 are classified as anomalies, while those below 0.5 are considered normal (28).

A 10-fold cross-validation procedure was set up using the caret package (30), allowing for robust hyperparameter tuning. A set of hyperparameters for the Isolation Forest model were defined using a grid, with sample size (256, 500, and 1,000) and number of trees (100, 200, and 300). Package *purrr* (31) was used to iterate through each combination of hyperparameters, training and evaluating the model on the training data and calculating the mean anomaly score for each fold. During cross-validation, the average anomaly score across folds was used as a relative performance metric for model selection. While this metric does not directly measure detection accuracy, it provides a consistent basis to compare configurations in the absence of a large labeled dataset. The small number of labeled anomalies was later used to evaluate the models' ability to flag known health-related anomalies, while also assessing their potential to uncover hidden or emerging anomalies. After cross-validation, the optimal hyperparameters, determined by the lowest average anomaly score, were selected, with a sample size equal to 700 and a number of trees equal to 100 (Table 1). The final model was trained with the best hyperparameters, and anomaly scores were predicted. Finally, anomaly scores were compared to a threshold using the 75th percentile of the anomaly scores to classify points as anomalies. The thresholding step was applied *post hoc* to the final trained model, rather than during cross-validation. Cross-validation was used exclusively for hyperparameter tuning, while the 75th percentile cutoff was applied once to the anomaly scores from the fully trained model. This separation ensured that thresholding did not influence model selection, but rather served as the final classification step. This approach used the *isotree* package (32) for anomaly detection. Using a threshold for anomaly detection has been an approach used in the literature (33). By using the 75th percentile as a threshold for the anomaly scores, it is possible to detect the most extreme outliers without being overly restrictive or lenient. This choice provided a simple and interpretable threshold that could be applied consistently across models. Percentile-based thresholding is commonly used in unsupervised settings where labeled anomalies are limited, as it allows for empirical selection of a cutoff based on the observed distribution of anomaly scores (34–36).

**Table 1:** Parameter values for the different models, or each production system.

**Production system**	**Model**	Parameters^*^	**Value**
**Production system 1**	Isolation Forest	Sample size	700
		Number of trees	100
	Autoencoder	Batch size	128
		Epochs	50
		Hidden layer 1	64
		Hidden layer 2	64
	KNN	k	3
**Production system 2**	Isolation Forest	Sample size	800
		Number of trees	300
	Autoencoder	Batch size	256
		Epochs	50
		Hidden layer 1	64
		Hidden layer 2	32
	KNN	k	3

^*^Sample size—Number of data points sampled to build each individual tree in the forest

Number of trees—Number of decision trees in the forest

Batch size—Number of training samples used in the model's forward/backward pass. It controls the amount of data fed into the model in each training iteration.

Epochs—Number of complete passes through the entire training dataset.

Hidden layer 1—Number of neurons in the first hidden layer of the autoencoder. This controls the complexity of the model and the capacity to capture patterns from the data.

Hidden layer 2—Number of neurons in the second hidden layer of the autoencoder. It controls the model's capacity to learn complex patterns.

k—Number of nearest neighbors to consider when making a classification or regression decision.

### K-nearest neighbor

K-nearest neighbor (KNN), a clustering technique, can be also implemented for anomaly detection with unsupervised learning. The principle behind this model is to find k predefined number of training samples that are closest in the distance to a new point and predict a label for the new point using the samples (37). The anomaly score [0, ∞] is calculated as follows:


$$\begin{array}{l} kNN\left(xq,\;X,\;k\right)=\;\frac{1}{k}\sum_{x\in Nk\;\left(X,\;xq\right)}||xq-x|\;|2, \end{array}$$

where *Nk(X, xq)* is the set of k nearest neighbors of xq, *xq* is the query data, and X is the search space (38).

In this study, a KNN algorithm was employed for anomaly detection using the *kknn* (39), and *FNN* (40) packages in R. First, a parameter grid was defined for tuning the number of neighbors (k), ranging from 3 to 10. A 10-fold cross-validation was used to evaluate different configurations, with each fold generating an anomaly score based on the sum of distances to the k-nearest neighbors. The KNN model was trained, utilizing a Euclidean distance (specified via the distance = 2 argument) and a rectangular kernel to compute the distances. The anomaly scores for each fold were computed as the row sums of these distances, representing the degree of “anomalousness” of each data point. The results of each fold were stored and averaged across all folds to identify the optimal hyperparameter (k) based on the lowest average anomaly score. After selecting the best k (k = 3), the final model was trained on the entire training dataset (Table 1). The FNN package was then used to calculate the final distances, with anomaly scores derived from the summed distances to the nearest neighbors. To classify anomalies, the 75th percentile of the anomaly scores was used as a threshold, and data points above this threshold were labeled as anomalies (33–36).

### Autoencoders

Autoencoders, a type of neural network, learn to compress and reconstruct input data by minimizing the reconstruction error or anomaly score; anomalies are detected based on poor reconstruction performance (the difference between the input and the output), as anomalies do not fit the learned patterns (22). The reconstruction error or anomaly score [0, ∞] is calculated as follows:


$$\begin{array}{l} \text{S}\left(\text{x}\text{i}\right)=||\text{x}i-\text{g}\text{u}\left(\text{f}\text{w}\left(\text{x}\text{i}\right)\right)||, \end{array}$$

where *xi* is the input data, *gu* is the decoder function, *fw* is the encoder function. Anomaly detection using autoencoders operates on the premise that anomalies are linked to higher values of *S(xi)*, reconstruction losses, while normal data is associated with lower reconstruction losses (22).

A 10-fold cross-validation procedure was set up allowing for hyperparameter tuning. A grid of hyperparameters was defined, including various configurations for the number of hidden units in the two layers, hidden layer 1 (64, 128, 256), and hidden layer 2 (32, 64), the number of epochs (50, 100), batch size (128, 256), and learning rate (0.001, 0.0001). An autoencoder model with the specified hyperparameters, using the Tanh activation function, L1/L2 regularization, and other training parameters, such as adaptive learning rates and mini-batch size, was created. The reconstruction error for each data point was calculated by comparing the original data to the model's reconstructed data. The reconstruction error was used to identify anomalies by setting a threshold based on the 75th percentile of the errors. A grid search was then performed to evaluate each combination of hyperparameters, and the average reconstruction error was calculated for each configuration. The best-performing hyperparameters were selected based on the lowest average reconstruction error (Table 1). The final autoencoder model hyperparameters had an average reconstruction error of 298.62. Reconstruction error scores were predicted, and anomalies were identified by comparing their reconstruction errors to the 75th percentile threshold (33–36). Throughout the process, functions from the *h2o* package (41) were utilized for model training, prediction, and evaluation, while base R functions were employed for data manipulation and grid search management.

### Assessing differences among anomalous and non-anomalous weeks

#### Permutation test to compare means

Given the non-normality, lack of homogeneity of variances, and potential dependence among observations in the data, a permutation test was used to compare the means of the productivity indicators between anomalous and non-anomalous weeks. The data are correlated by subject over time, so permutation was performed by reshuffling the subjects, ensuring that all observations within the same subject were resampled together. Permutation tests are a non-parametric alternative to traditional hypothesis tests and do not rely on assumptions of normality, homoscedasticity, or independence. By reshuffling the observed data at the subject level and recalculating the test statistic for each permutation, permutation tests create a distribution of the test statistic under the null hypothesis. This process enables the estimation of a *p*-value without making assumptions about the underlying distribution of the data (42). A summary table was created to compare anomalous and non-anomalous weeks with the average production indicator values, reconstruction errors, and anomaly scores. The same process was done according to each source of the health challenge. The health challenge sources were defined according to the outbreak report, and if there was no outbreak report, the anomalies were defined as “Others.” PRRSV and SVV were considered true outbreaks, and everything else was considered as no outbreak or “Others.”

#### Density plots

Density plots were built for each productivity indicator for each model and production system. The density plots display the distribution of the log_10_ values of productivity indicators, with the *x*-axis representing the log-transformed productivity values and the *y*-axis representing the density, or the relative frequency, of those values. In the plot, two sets of curves are shown: the red curves represent the anomalous weeks, while the blue curves represent the non-anomalous weeks. By comparing the density of the red and blue curves, one can observe how the distribution of productivity indicators differ between anomalous and non-anomalous weeks.

### Performance

A confusion matrix was created to calculate the performance. Binary labels for both predicted anomalies and true anomalies were defined. True anomalies were considered only for the weeks with health challenge labels (PRRSV and SVV), and these were marked as anomalies (1). In Production System 1, 152 weeks were labeled as PRRSV, while in Production System 2, 821 weeks were labeled as PRRSV, and 72 weeks were labeled SVV. Weeks that were not associated with health challenges were not considered true anomalies and were classified as negative (0). In Production System 1, this included 1,192 weeks labeled as “Others” (0), and in Production System 2, 5,807 weeks were classified as “Others” (0). A TP was considered when there was an anomaly (1), and the models identified an anomalous week. A TN was considered when there was no anomaly (0), and the models did not identify an anomalous week. An FP was considered when there was no anomaly in a specific week, but the models identified that week as being an anomalous week. Lastly, an FN was considered when there was an anomaly in a specific week (1), but the models did not identify that week as being anomalous. Using the confusion matrix, precision (the proportion of true positives out of all predicted positives), recall (the proportion of true positives out of all actual anomalies), and F1-score (the harmonic mean of precision and recall) were calculated. The F1-score, which balances both precision and recall, was used to evaluate the overall performance of the models.

### PRRSV example line plots

Since the dataset had a total of 973 weeks with PRRSV outbreaks, line plots were created for the 8 weeks before and 15 weeks following an outbreak for each production system. These plots illustrated the deviations in productivity indicators during anomalous weeks, potentially associated with PRRSV outbreaks, compared to non-anomalous weeks.

## Results

### Descriptive

A total of 8,044 weeks were analyzed. The total number of anomalous weeks for Production System 1 was 336 and the number of non-anomalous weeks was 1,008. For Production System 2, the number of anomalous weeks was 1,675, and the number of non-anomalous weeks was 5,025.

### Production System 1

In Production System 1, the differences between anomalous and non-anomalous weeks across the three anomaly detection models (Isolation Forest, Autoencoder, and KNN) revealed that abortions per 1,000 sows had a significant increase in anomalous weeks (*p*-value <0.001) for all three models, with a difference in means ranging from 1.70 (Autoencoder) to 2.95 (Isolation Forest), suggesting that anomalous weeks are associated with a higher number of abortions. Prenatal losses also displayed significant differences, with Isolation Forest and KNN showing the largest effects (difference of 0.84 with *p*-value 0.01 and difference of 0.93 with *p*-value <0.001, respectively), indicating that anomalous weeks had increased prenatal losses. Conversely, total born and liveborn both showed negative differences in means (with Isolation Forest reporting a difference of −1.48 and a difference of −1.75 for total born and liveborn, with *p*-values <0.001, respectively), pointing to lower birth rates in anomalous weeks. The sows culled per 1,000 sows showed a higher difference for anomalous weeks (e.g., 12.27 with *p*-value <0.001 for KNN) than for non-anomalous weeks. Lastly, the anomaly scores revealed large differences (e.g., 1,677.60 for Autoencoder with *p*-value <0.001), emphasizing that weeks with anomalies exhibit a highly elevated anomaly score compared to non-anomalous weeks (Table 2).

**Table 2:** Permutation test results for production system 1 and production system 2.

**Production system**	**Variable**	**Isolation forest**		**Autoencoder**		**KNN**	
		**Difference in means** ^*^	***p*** **-Value**	**Difference in means** ^*^	***p*** **-Value**	**Difference in means** ^*^	***p*** **-Value**
1	Abortions per 1,000	2.95	<0.001	1.70	<0.001	2.72	<0.001
	Prenatal losses	0.84	0.01	0.34	0.32	0.93	<0.001
	Preweaning mortality	0.01	0.08	0.01	0.35	0.01	0.09
	Total born	−1.48	<0.001	−1.09	<0.001	0.31	0.31
	Liveborn	−1.75	<0.001	−1.31	<0.001	−0.15	0.61
	Sow culled per 1,000	10.04	0.02	10.79	<0.001	12.27	<0.001
	Sow deaths per 1,000	0.96	<0.001	0.00	1.00	1.01	<0.001
	Anomaly score	0.12	<0.001	1,677.60	<0.001	67.69	<0.001
2	Abortions per 1,000	4.12	<0.001	3.08	<0.001	4.56	<0.001
	Prenatal losses	0.79	<0.001	0.41	<0.001	0.70	<0.001
	Preweaning mortality	7.35	<0.001	8.81	<0.001	8.87	<0.001
	Total born	−1.12	<0.001	−1.19	<0.001	−0.13	0.25
	Liveborn	−1.92	<0.001	−1.60	<0.001	−0.84	<0.001
	Sow culled per 1,000	27.59	<0.001	29.79	<0.001	38.18	<0.001
	Sow deaths per 1,000	1.45	<0.001	1.22	<0.001	2.00	<0.001
	Anomaly score	0.11	<0.001	59,177.13	<0.001	60.32	<0.001

^*^The difference in means displayed in the table reflects the difference between anomalous and non-anomalous weeks, calculated using a permutation test (Anomalous - Non-anomalous)

When analyzing the weeks classified as having PRRSV, we see significant differences in many productivity indicators. The abortions per 1,000 sows showed differences across all models, with a large difference in means, particularly in Isolation Forest (3.90, *p*-value <0.001), followed by KNN (3.91, *p*-value <0.001), indicating an increase in abortions in anomalous weeks. Significant differences were also observed in total born across all models (*p*-value <0.01) and liveborn for Isolation Forest and Autoencoder (*p*-value <0.001), with negative differences suggesting lower numbers in anomalous weeks. Sows culled per 1,000 sows showed an increase in the number of culled sows during anomalous weeks, particularly with the KNN model (15.38, *p*-value = 0.01). Anomaly scores demonstrated high values in anomalous weeks associated with PRRSV, especially in the Autoencoder model (2,274.80). The weeks classified as “Others” showed less drastic differences in most variables, but sows culled per 1,000 sows and anomaly scores remained significant across all models with *p*-values <0.001 (Table 3).

**Table 3:** Permutation test results for production system 1 and production system 2 for different anomalies.

**Production system**	**Anomaly type**	**Variable**	**Isolation Forest**		**Autoencoder**		**KNN**	
			**Difference in means** ^*^	***p*** **-Value**	**Difference in means** ^*^	***p*** **-Value**	**Difference in means** ^*^	***p*** **-Value**
1	PRRSV	Abortions per 1,000	3.90	<0.001	1.86	0.05	3.91	<0.001
		Prenatal losses	0.44	0.44	0.46	0.43	0.96	0.08
		Preweaning mortality	0.00	0.60	0.01	0.43	0.01	0.33
		Total born	−1.73	<0.001	−1.50	0.01	1.05	0.07
		Liveborn	−2.12	<0.001	−1.74	<0.001	0.45	0.39
		Sow culled per 1,000	11.60	0.38	14.17	0.06	15.38	0.01
		Sow deaths per 1,000	1.21	0.01	0.13	0.77	1.66	<0.001
		Anomaly score	0.13	<0.001	2,274.80	0.47	91.38	<0.001
	Others	Abortions per 1,000	0.08	0.43	−0.01	0.95	0.36	0.04
		Prenatal losses	1.58	<0.001	−0.03	0.95	0.78	0.05
		Preweaning mortality	0.04	<0.001	0.00	0.89	0.01	0.27
		Total born	−0.79	<0.001	−0.09	0.56	−0.23	0.10
		Liveborn	−0.74	<0.001	−0.16	0.25	−0.46	<0.001
		Sow culled per 1,000	5.73	<0.001	4.22	0.01	8.39	<0.001
		Sow deaths per 1,000	0.55	0.05	−0.53	0.05	0.17	0.57
		Anomaly score	0.07	<0.001	47.95	<0.001	20.00	<0.001
2	PRRSV	Abortions per 1,000	5.20	<0.001	4.32	<0.001	6.07	<0.001
		Prenatal losses	1.05	<0.001	0.51	<0.001	0.82	<0.001
		Preweaning mortality	9.60	<0.001	12.57	<0.001	10.53	<0.001
		Total born	−1.01	<0.001	−1.18	<0.001	0.13	0.49
		Liveborn	−2.06	<0.001	−1.70	<0.001	−0.69	<0.001
		Sow culled per 1,000	29.62	0.02	39.15	<0.001	39.52	<0.001
		Sow deaths per 1,000	1.64	<0.001	1.63	<0.001	2.28	<0.001
		Anomaly score	0.12	<0.001	90,289.09	<0.001	66.56	<0.001
	Others	Abortions per 1,000	0.95	<0.001	0.26	0.06	0.73	<0.001
		Prenatal losses	0.02	0.66	−0.05	0.21	0.10	0.01
		Preweaning mortality	2.32	<0.001	2.69	<0.001	4.72	<0.001
		Total born	−1.40	<0.001	−1.22	<0.001	−0.08	0.67
		Liveborn	−1.42	<0.001	−1.18	<0.001	−0.17	0.25
		Sow culled per 1,000	22.31	<0.001	15.25	<0.001	37.47	<0.001
		Sow deaths per 1,000	0.70	<0.001	0.35	0.03	1.18	<0.001
		Anomaly score	0.08	<0.001	9,968.82	<0.001	46.33	<0.001
	SVV	Abortions per 1,000	0.48	0.06	0.00	0.99	0.51	0.05
		Prenatal losses	−0.12	0.28	0.13	0.19	−0.02	0.83
		Preweaning mortality	2.35	0.45	3.04	0.27	7.47	0.03
		Total born	−0.09	0.73	0.64	<0.001	0.26	0.35
		Liveborn	0.04	0.88	0.51	0.01	0.28	0.24
		Sow culled per 1,000	8.51	0.01	−1.49	0.61	16.52	<0.001
		Sow deaths per 1,000	0.89	0.03	−0.07	0.83	0.70	0.11
		Anomaly score	0.05	<0.001	265.32	<0.001	19.80	<0.001

PRRSV, Porcine reproductive and respiratory syndrome virus; SVV, Seneca Valley virus.

*The difference in means displayed in the table reflects the difference between anomalous and non-anomalous weeks, calculated using a permutation test (Anomalous - Non-anomalous).

**Figure 1:**
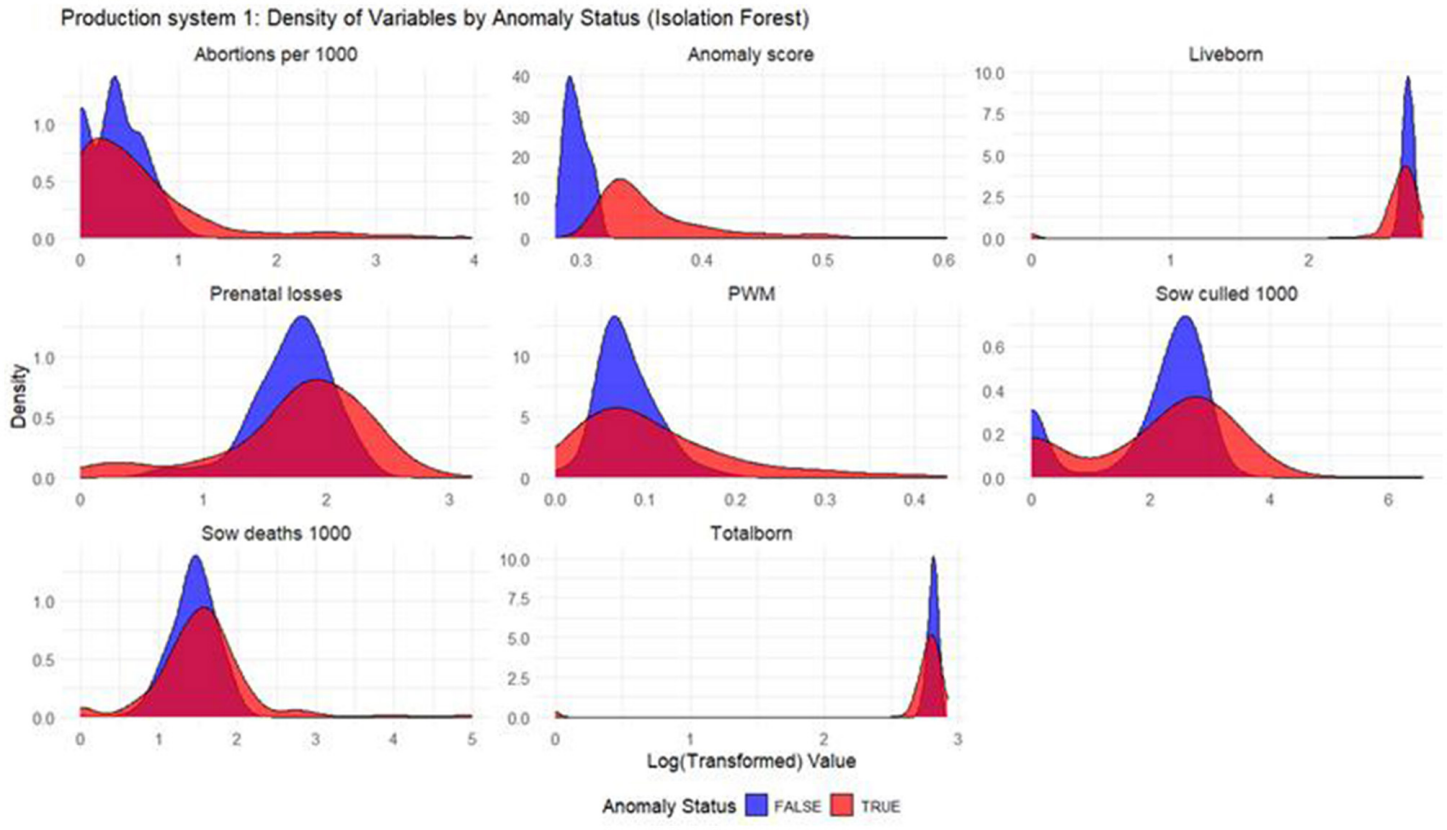
Density plots for Isolation Forest (Production System 1). Each production indicator and the anomaly scores are shown in the density plots; the y-axis represents the density, and the x-axis represents the log value of each variable (the log transformation was done for better interpretation of the chart). The blue color represents the non-anomalous weeks, and the red color represents the anomalous weeks. The plots illustrate how the model separates non-anomalous and anomalous weeks, providing a visual overview of deviations in production indicators relevant for anomaly detection.

**Figure 2:**
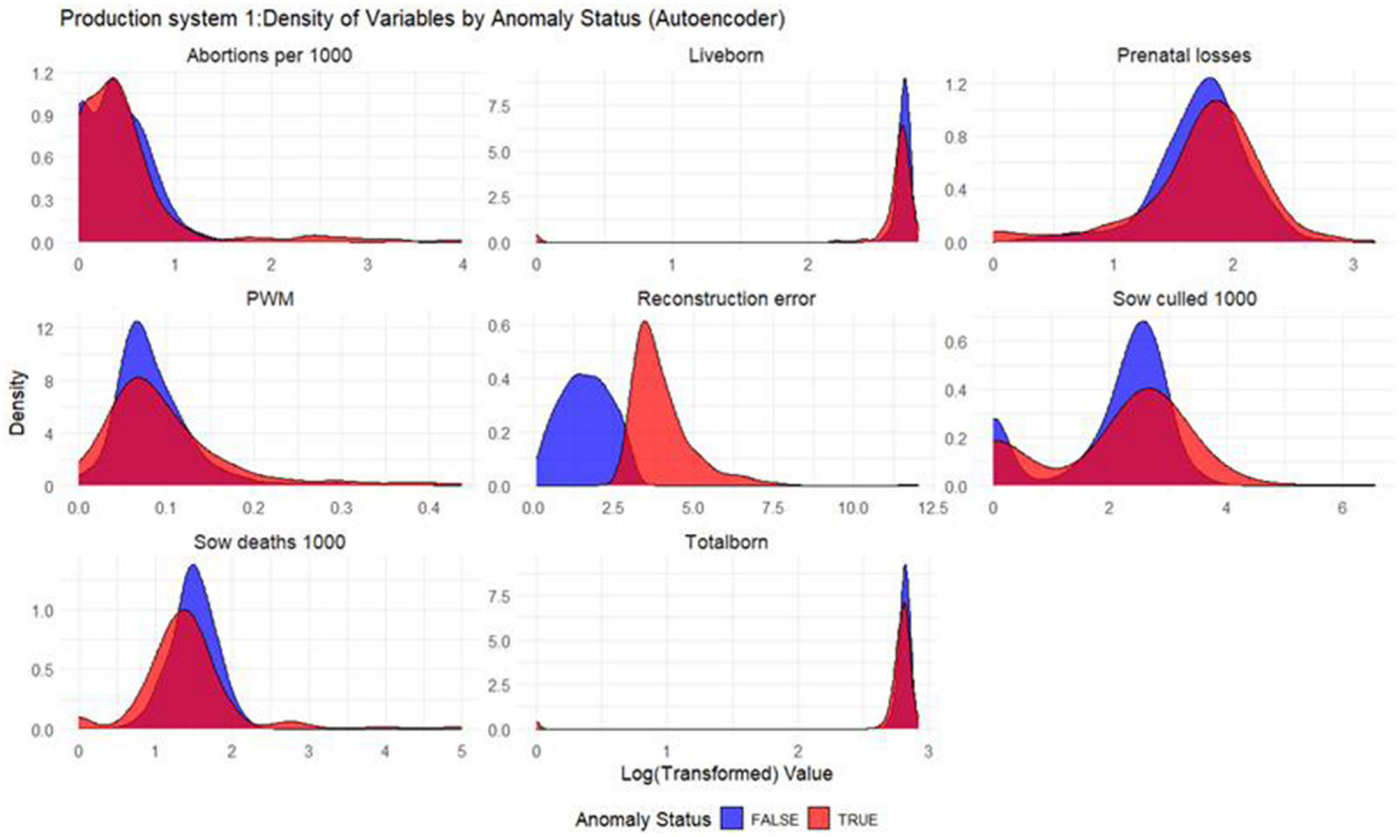
Density plots for Autoencoder (Production System 1). Each production indicator and the reconstruction error are shown in the density plots; the y-axis represents the density, and the x-axis represents the log value of each variable (the log transformation was done for better interpretation of the chart). The blue color represents the non-anomalous weeks, and the red color represents the anomalous weeks. The plots illustrate how the model separates non-anomalous and anomalous weeks, providing a visual overview of deviations in production indicators relevant for anomaly detection.

**Figure 3:**
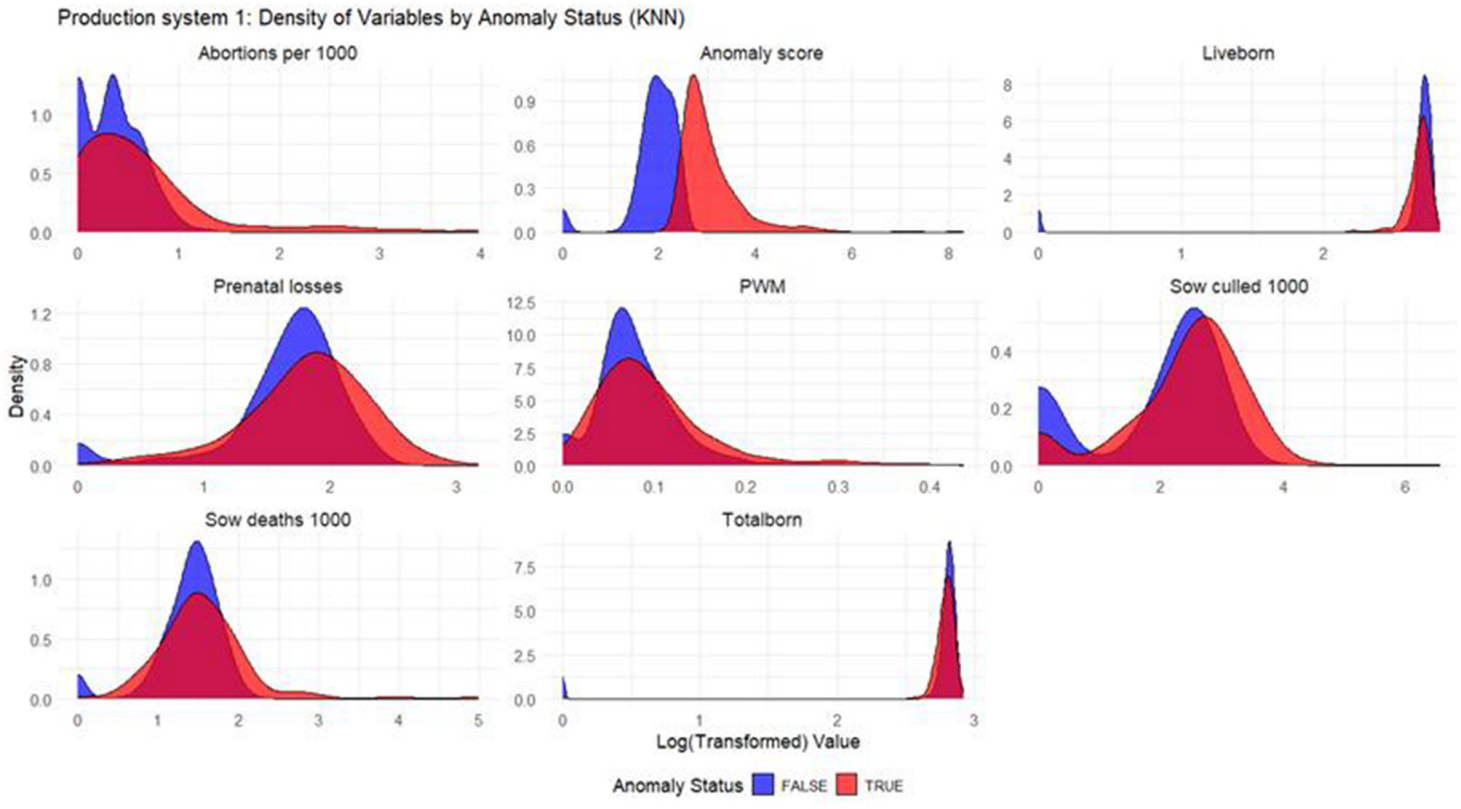
Density plots for KNN (Production System 1). Each production indicator and the anomaly score are shown in the density plots; the y-axis represents the density, and the x-axis represents the log value of each variable (the log transformation was done for better interpretation of the chart). The blue color represents the non-anomalous weeks, and the red color represents the anomalous weeks. The plots illustrate how the model separates non-anomalous and anomalous weeks, providing a visual overview of deviations in production indicators relevant for anomaly detection.

Based on the density plots for Isolation Forest (Figure 1), Autoencoders (Figure 2), and KNN (Figure 3), it can be concluded that, overall, productivity indicators such as abortions per 1,000 sows and sows culled per 1,000 sows during anomalous weeks (marked in red) tend to be higher than those in non-anomalous weeks (marked in blue). This is evident as the tails of the density curve for anomalous weeks stretch far to the right side of the plot for these productivity indicators, indicating higher values. In contrast, for liveborn and total born, the tails of the density curve for anomalous weeks reach far to the left, signifying lower values compared to non-anomalous weeks.

### Production System 2

In Production System 2, the differences between anomalous and non-anomalous weeks remain consistent across the three models. Abortions per 1,000 sows showed significant increases for anomalous weeks, with the difference in means ranging from 3.08 (Autoencoder) to 4.56 (KNN), with *p*-values <0.001. Prenatal losses showed smaller but significant differences across all methods, with Isolation Forest reporting the highest effect (0.79, *p*-value <0.001). Like Production System 1, total born and liveborn both showed negative differences, with total born decreasing by as much as −1.12, with *p*-value <0.001 (Isolation Forest), indicating reduced birth rates for anomalous weeks. Sows culled per 1,000 sows showed large differences between anomalous and non-anomalous weeks, with values ranging from 27.59 (Isolation Forest) to 38.18 (KNN), with *p*-value <0.001, suggesting increased sow culling in this production system during anomalous weeks. The anomaly scores showed a disparity between anomalous and non-anomalous weeks, with Autoencoder reporting a high difference of 59,177.13 with a *p*-value <0.001 (Table 2).

For the weeks classified as PRRSV, these exhibited significant differences, particularly in abortions per 1,000 sows, which showed differences in means across models (ranging from 4.32 for Autoencoder to 6.07 for KNN, with *p*-value <0.001), indicating a high number of abortions in anomalous weeks associated with a PRRSV. Similarly, PWM showed significant differences, with high values in anomalous weeks across models (*p*-value <0.001). Sows culled per 1,000 sows showed high differences in the number of culled sows, particularly with the KNN model (39.52, *p*-value <0.001). Significant differences in anomaly scores were observed across all models, with extremely high values for PRRSV anomalies, especially in the Autoencoder model (90,289.09, *p*-value <0.001). The weeks classified as SVV presented more variability, with less consistent differences, particularly in prenatal losses and liveborn. This likely reflects the milder and short-lived clinical expression of SVV in sows, as infection typically resolves within 8–10 days and does not severely affect reproductive parameters, unlike PRRSV (27). However, sows culled 1,000 sows still showed significant differences, with large values for the KNN model (16.52, *p*-value <0.001). When comparing the weeks classified as “Others,” significant differences in variables like abortions per 1,000 sows (Isolation Forest: 0.95, *p*-value <0.001; Autoencoder: 0.26, *p*-value = 0.06; KNN: 0.73, *p*-value <0.001 ) and sows culled per 1,000 sows (Isolation Forest: 22.31, *p*-value <0.001; Autoencoder: 15.25, *p*-value <0.001; KNN: 37.47, *p*-value <0.001 ) were observed, but with smaller values than those associated with PRRSV (Table 3).

**Figure 4:**
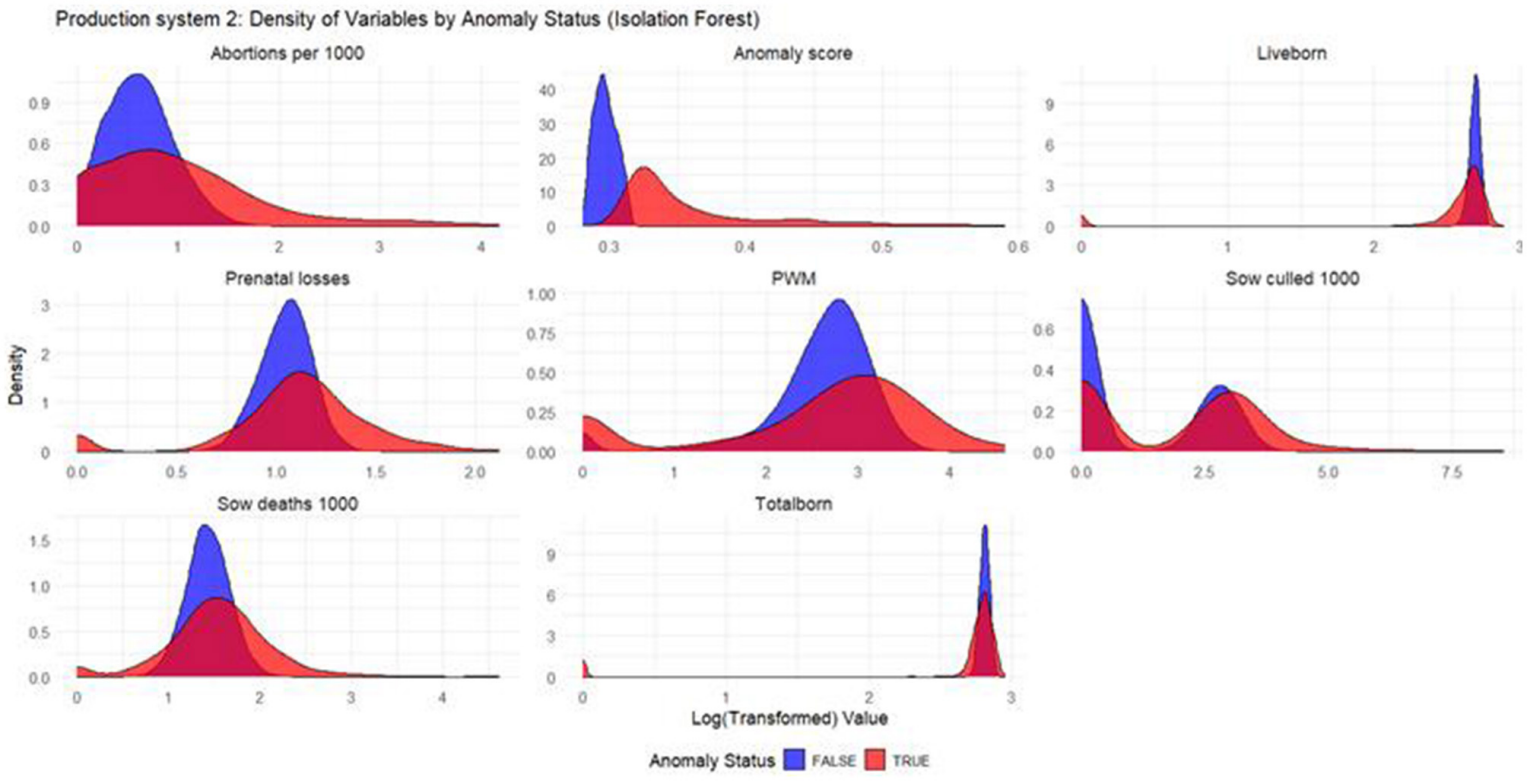
Density plots for Isolation Forest (Production System 2). Each production indicator and the anomaly scores are shown in the density plots; the y-axis represents the density, and the x-axis represents the log value of each variable (the log transformation was done for better interpretation of the chart). The blue color represents the non-anomalous weeks, and the red color represents the anomalous weeks.

**Figure 5:**
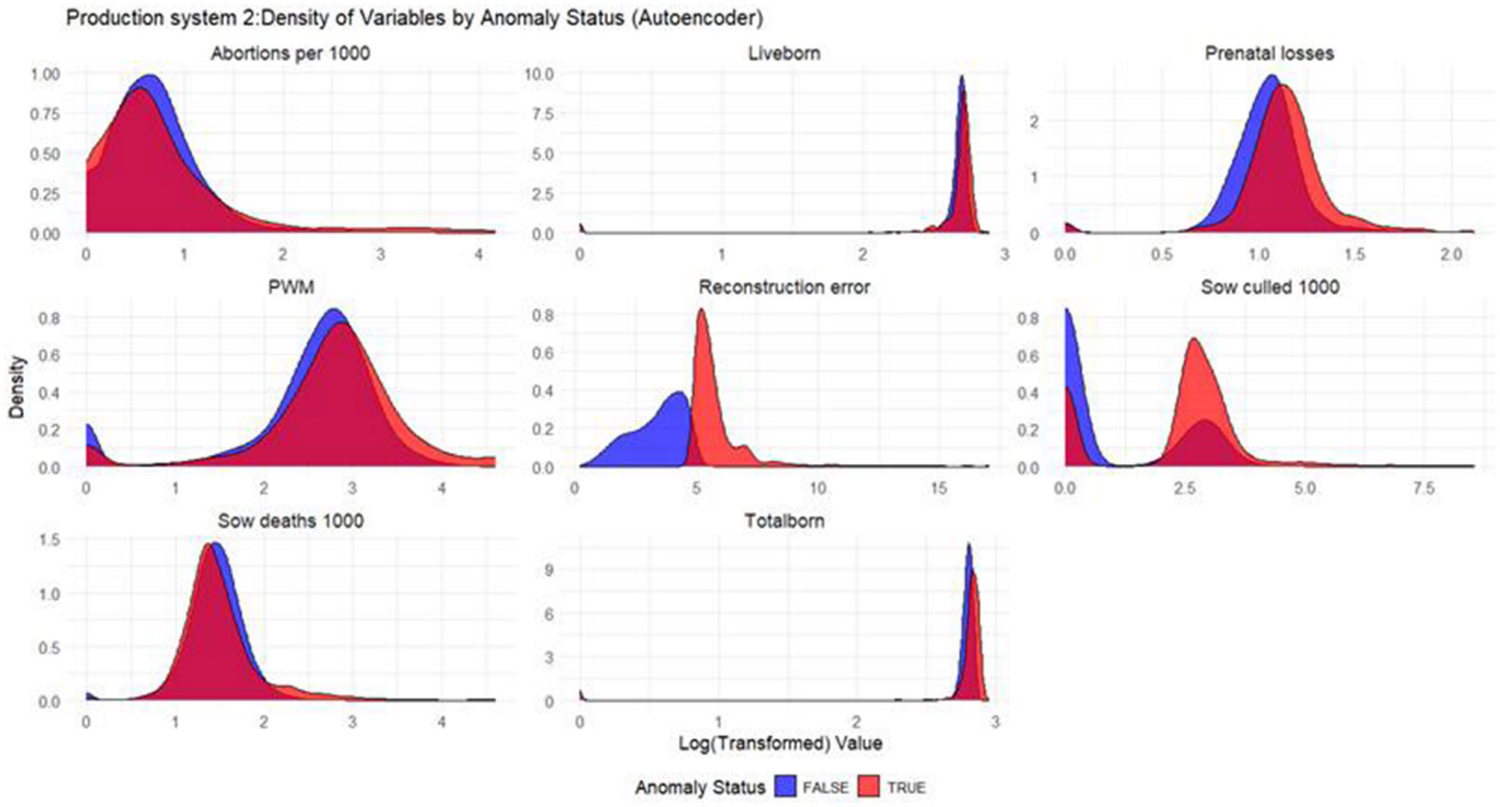
Density plots for Autoencoder (Production System 2). Each production indicator and the reconstruction error are shown in the density plots; the y-axis represents the density, and the x-axis represents the log value of each variable (the log transformation was done for better interpretation of the chart). The blue color represents the non-anomalous weeks, and the red color represents the anomalous weeks.

**Figure 6:**
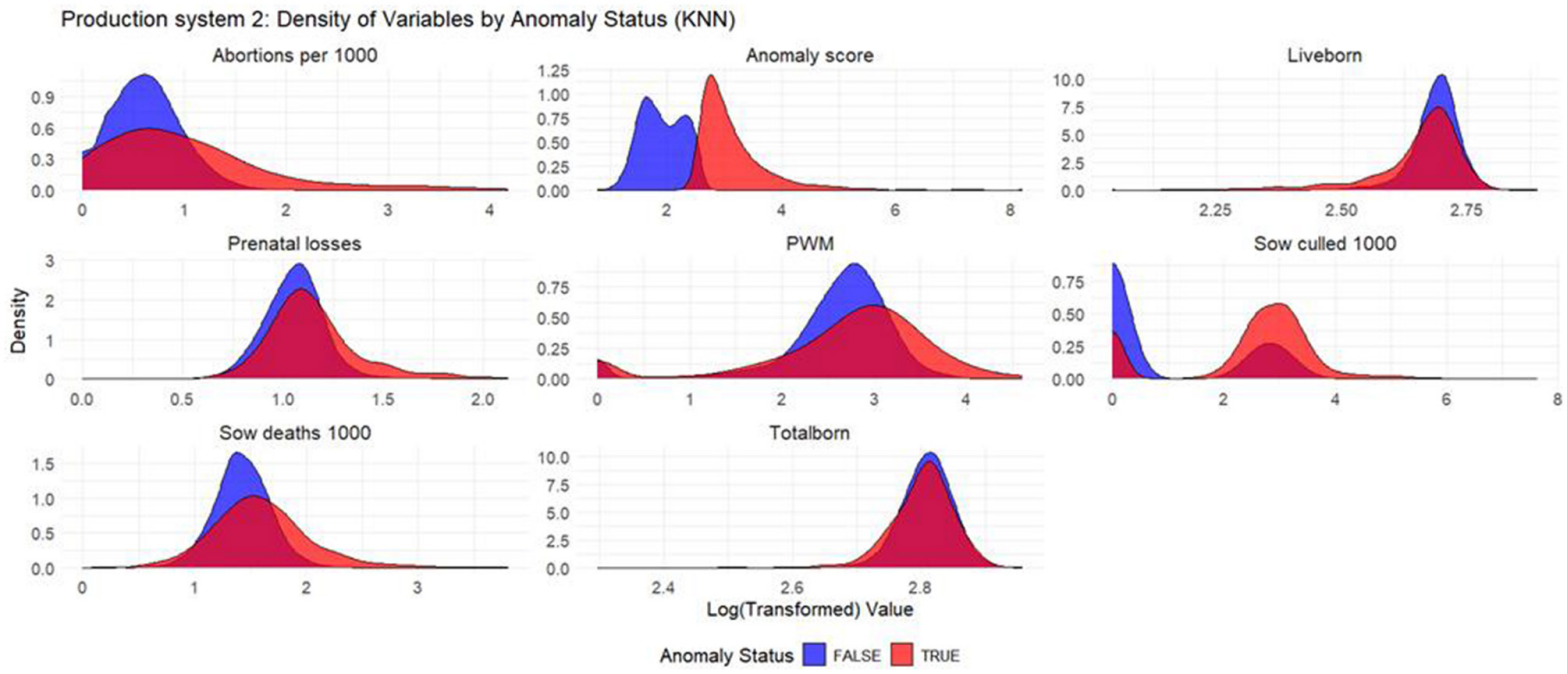
Density plots for KNN (Production System 2). Each production indicator and the anomaly score are shown in the density plots; the y-axis represents the density, and the x-axis represents the log value of each variable (the log transformation was done for better interpretation of the chart). The blue color represents the non-anomalous weeks, and the red color represents the anomalous weeks.

Based on the density plots for Isolation Forest (Figure 4), Autoencoders (Figure 5), and KNN (Figure 6), the conclusions from the charts are like those for Production System 1.

### Performance

The models performed well in detecting the PRRSV anomaly, achieving perfect precision (100%) across all methods and for both production systems, with F1-scores ranging from 60% (Autoencoder for Production System 2) to 77% (Isolation Forest for Production System 2). For anomalies like SVV the models showed lower F1-scores for the Isolation Forest and the Autoencoder models compared to PRRSV (Table 4).

**Table 4:** Performance metrics per anomaly type for each production system.

**Production system**	**Anomaly type**	**Isolation Forest**			**Autoencoder**			**KNN**		
		**Precision**	**Recall**	**F1-score**	**Precision**	**Recall**	**F1-score**	**Precision**	**Recall**	**F1-score**
1	PRRSV	100%	51%	67%	100%	52%	68%	100%	50%	67%
2	PRRSV	100%	62%	77%	100%	43%	60%	100%	52%	68%
	SVV	100%	13%	22%	100%	18%	31%	100%	50%	67%

### PRRSV example line plots

**Figure 7:**
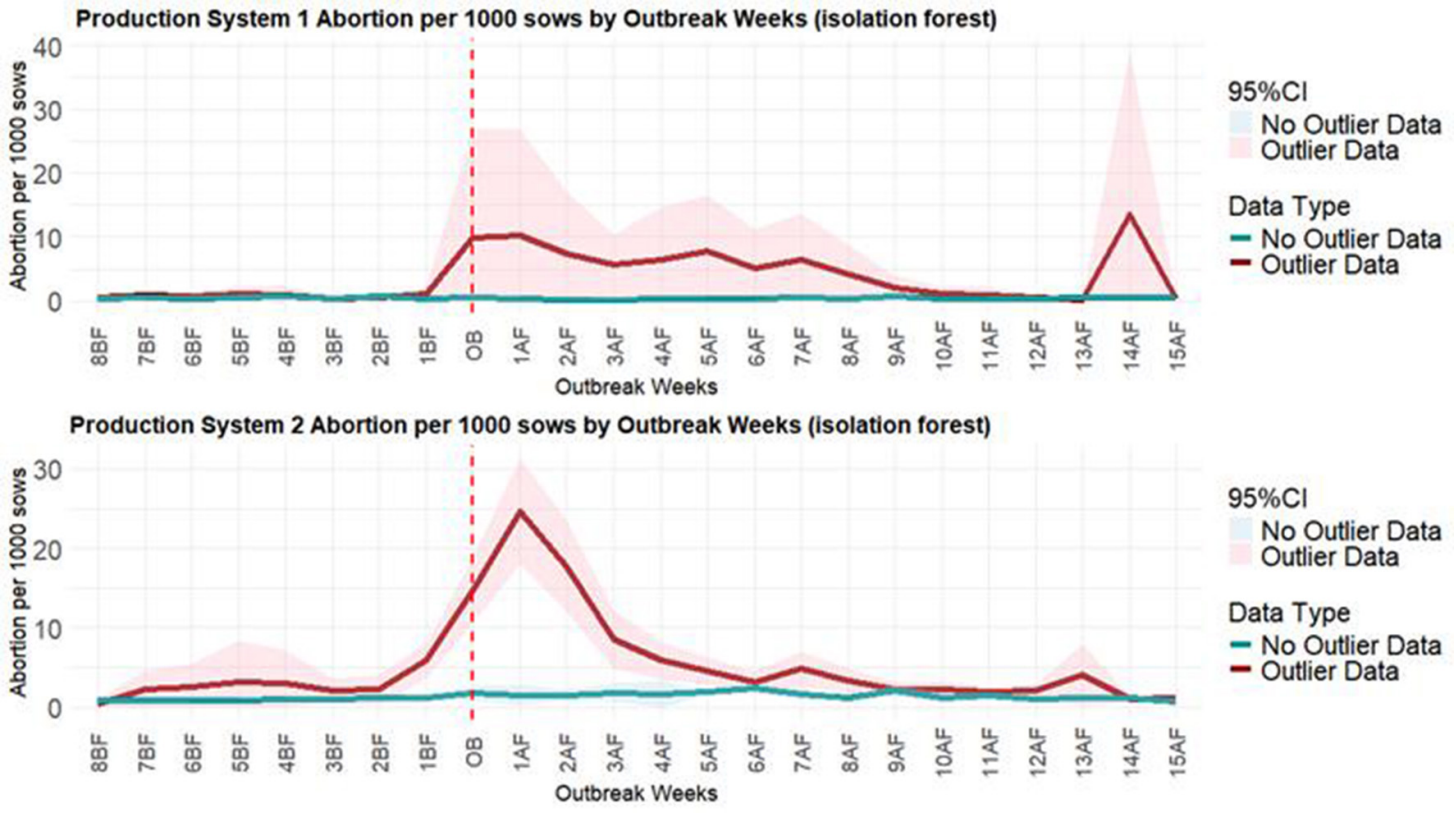
The average of abortions per 1,000 sows in the weeks prior to and following PRRSV outbreaks. The chart on the top refers to the isolation forest model with the average of abortions per 1,000 sows for Production System 1. The chart on the bottom refers to the isolation forest model with the average of abortions per 1,000 sows for Production System 2. The red line is the average for anomalous weeks, and the green line is the average for non-anomalous weeks. The vertical dots refer to the week of the PRRSV outbreak.

The PRRSV line plots (Figure 7) illustrate the differences between weeks classified as anomalous due to a PRRSV outbreak and those that were not considered anomalous. It can be observed that the number of abortions per 1,000 sows began to rise before the diagnosis of the PRRSV outbreak, especially noticeable in the line plot for Production System 2, and that the number of abortions reached higher values than those reached during the non-anomalous weeks.

## Discussion

This paper evaluates the use of unsupervised machine learning models to detect anomalies in productivity indicators on swine farms. Given the increasing automation in data collection and analysis in modern swine production systems, leveraging this data is crucial for monitoring health status and improving farm management (43, 44). These systems collect large volumes of data on production performance, which could be analyzed to identify health-related anomalies. Anomaly detection techniques, such as those explored in this study, can provide early warnings for diseases, enabling timely interventions and enhancing productivity monitoring.

Previous research has explored anomaly detection in the context of animal health and health challenges. For example, gated recurrent units -Autoencoders have been used to detect early signs of respiratory diseases in growing-pig farms, focusing on environmental factors (57). Additionally, a study by **(author?)** (58) found that the long short-term memory Autoencoder model outperformed other models, including Farrington, Early Aberration Reporting System, and regression models, in detecting anomalies related to PRRSV. Similarly, the Isolation Forest model, previously used to detect estrus in dairy cows (59) and to identify anomalies in temperature and humidity data (60), performed well in distinguishing between anomalous and non-anomalous weeks. The KNN model, known for its effectiveness in both supervised and unsupervised learning (61, 62), showed similar results to the Autoencoder and Isolation Forest models in this study, with anomalous weeks showing significant differences between anomalous and non-anomalous weeks (Table 2).

The models employed in this study represent distinct anomaly detection approaches, each with its strengths and limitations. Isolation Forest is known for its efficiency, scalability, and ability to detect anomalies without assumptions about data distribution, making it suitable for high-dimensional data (28, 29). However, its performance may decline with highly imbalanced datasets or when anomalies closely resemble normal instances (45). In contrast, Autoencoders use neural networks to capture complex, non-linear relationships within high-dimensional datasets, making them effective for detecting subtle anomalies (22). However, they can be computationally intensive and require careful tuning to avoid issues related to training anomalies and generalization (45). KNN, a non-parametric model that requires minimal parameters, is versatile for both classification and regression tasks. However, it struggles with high-dimensional data and is prone to overfitting (46). Despite these limitations, all models provided valuable insights into production behavior during anomalous and non-anomalous weeks (Table 2).

In the case of disease detection, it is of the utmost importance to guarantee that possible epidemics are caught early. Previous studies using statistical process control charts observed that deviations in productivity indicators start to be noticed on average 4 weeks before the diagnostic confirmation of a PRRSV outbreak (8, 10). The results from this study showed that the Isolation Forest, KNN, and Autoencoder models indicated a trend of increasing number of anomalies in productivity indicators associated with outbreaks compared to weeks that were not considered anomalies (Table 3). These findings underscore the complementary nature of the Isolation Forest, KNN, and Autoencoder models, providing valuable insights into how productivity indicators fluctuate during significant events and demonstrating their potential for monitoring and detecting anomalies in swine production systems. For instance, for both production systems, the KNN model detected a higher difference in abortions per 1,000 sows during PRRSV outbreaks (Production System 1: 3.91, Production System 2: 6.07) than the other two models. Conversely, for SVV outbreaks, less pronounced differences between anomalous and non-anomalous weeks were observed across the models, suggesting that SVV-related disruptions may cause less noticeable changes in production performance. These findings underscore the value of applying multiple anomaly detection models to capture a wide range of disease-related fluctuations in swine production. Each model may be sensitive to different types of anomalies, some may detect extreme deviations, while others may better identify subtler or multivariate patterns, so comparing results across models can help uncover patterns that might be missed by any single method (47). Although no formal ensemble or voting approach was tested in this study, evaluating multiple models in parallel provided complementary insights and highlighted the potential for integrating diverse anomaly detection approaches in future work, particularly when routine diagnostics are not implemented at the farm.

The analysis of anomalous and non-anomalous weeks, based on different anomaly classifications (PRRSV, SVV, and Others), reveals variations in the productivity indicators, which aligns with findings from several studies on swine production. For instance, PRRSV-infected herds have been reported to exhibit higher rates of abortion, prenatal loss, and mortality (48, 49), which is consistent with the higher differences in anomaly scores, reconstruction errors, and productivity indicators observed during anomalous weeks in this study (Table 3). Specifically, the increased anomalies in PRRSV-classified weeks suggest a disruption in normal reproductive performance, as demonstrated by the elevated production losses seen in the literature (23). Furthermore, similar trends were observed for SVV, where anomalous weeks showed deviations in productivity indicators, similar to the experimental findings of other studies (24, 50), who reported production losses following SVV infections. These findings also corroborate (26), who emphasized the need for greater surveillance of SVV due to its potential to cause significant production losses. In contrast, non-anomalous weeks, particularly those classified as “Others,” showed smaller differences in productivity indicators between anomalous and non-anomalous weeks (Table 3), reflecting the absence of major health challenges as noted in the literature for non-endemic diseases (48). Collectively, these results highlight the significant role that diseases play in negatively affecting the performance and overall health of swine herds, reinforcing the importance of targeted interventions for mitigating these viral infections.

The F1-score, precision and recall metrics were used to evaluate the performance of the unsupervised anomaly detection models tested in this study (7, 51, 52). These metrics yielded F1-Scores values below 80%, with PRRSV reaching higher performance scores than SVV. As observed by the results of the permutation tests, this was expected, since PRRSV showed a larger difference in means between anomalous and non-anomalous weeks than the SVV and “Others.” It should be taken into consideration that the performance metrics were calculated based on a limited set of labeled data, with only weeks associated with specific health challenges (PRRSV and SVV) considered as true anomalies. In the context of unsupervised learning, these models are particularly useful for exploring patterns in datasets where labeled anomalies are limited. By detecting deviations without relying solely on predefined labels, the models in this study were able to highlight weeks with statistically significant differences from non-anomalous weeks, as confirmed by permutation tests comparing productivity indicators. This suggests that unsupervised approaches can help reveal anomalies that may have been missed, undiagnosed, or mislabeled, providing insight into potentially overlooked issues within the production system. While supervised models depend on the availability and accuracy of labels, unsupervised models can flag unusual patterns even when diagnostic data are incomplete or uncertain. One practical way to leverage these insights is through the creation of production scores that summarize deviations across production systems or farms. For example, farms or production systems could be categorized based on overall performance scores derived from anomaly detection outputs, allowing managers to identify areas with major deviations and prioritize investigations. Such scoring frameworks can provide a structured and interpretable method to monitor production variability.

While this study provides valuable insights into anomaly detection for disease monitoring in swine farms, it is important to acknowledge its limitations. The analysis primarily focused on health challenges, particularly PRRSV and SVV, and did not consider other factors that may influence the appearance of anomalies, such as environmental conditions or management practices. These models are intended to be agnostic, that is, they flag deviations in productivity indicators without assuming a specific cause, serving as an exploratory tool to highlight potential issues rather than a definitive measure of disease presence. Some flagged anomalies may represent false positives, triggered by routine management activities (e.g., vaccination, moving sows, or issues with electronic sow feeders), environmental events (e.g., weather), or missing data inputs, while others could reflect undiagnosed or emerging health challenges. if data are not entered or if key variables show unexpected gaps, the models would likely identify this as an anomaly, since the resulting deviation from the established farm-specific pattern differs from the expected trajectory of production indicators. In this sense, the models can also serve as a tool to detect irregularities in data recording or reporting practices. However, they cannot distinguish between anomalies arising from biological events and those caused by data-entry errors, emphasizing the need for human interpretation of the alerts. In this study, a threshold using the 75th percentile was used, but thresholds may need to be tuned in each production system to balance sensitivity and specificity, for example, by adjusting cutoffs based on production system-specific baseline variability or by applying adaptive approaches that account for temporal changes. Furthermore, the study did not assess PRRSV statuses prior to outbreak reports, which could have impacted the results, as farms with different levels of prior exposure to PRRSV may exhibit varying responses. Another potential limitation of the data used in this study is that the data for the same herds across consecutive weeks may exhibit temporal correlation. For example, production indicators could be influenced by health or environmental factors persisting over time. However, the models used in this study focus on detecting local anomalies based on deviations from the learned distribution or neighborhood (53). These models are not explicitly designed to account for temporal correlations but are still effective at identifying significant deviations regardless of the relationship between weeks.

Future research could broaden the scope by considering additional factors like environmental conditions, pathogen introductions, or farm management differences, providing a more comprehensive understanding of anomaly detection in swine production systems. Moreover, exploring other farm settings, such as nurseries or wean-to-finish operations, could offer further insights into the robustness and applicability of the models tested in this study. Additionally, considering the incorporation of time-series models or temporal correlation-aware anomaly detection techniques could be used to capture the dependencies of production indicators over time and enhance the model performance (54).

## Conclusion

The utility of unsupervised machine learning techniques in detecting anomalies was demonstrated in this study, paving the way for more proactive management strategies in livestock health. By employing Isolation Forest, Autoencoder, and KNN models, it was found that anomalous weeks showed a significant difference in the overall values of the productivity indicators, anomaly scores, and reconstruction errors compared to non-anomalous weeks. These findings highlight the potential for using productivity indicators to aid decision-makers in implementing surveillance systems, mainly in farms that are not routinely collecting samples for diagnostics. Monitoring these data is crucial for triggering timely diagnostic tests and treatments and enhancing biosecurity measures, ultimately contributing to improved animal welfare and farm productivity during health challenges.

## Data Availability

The data that support the findings of this study belong to two production systems and restrictions apply to the availability of these data, which were used under license for the current study, and so are not publicly available. Data are, however, available from the authors upon reasonable request and with permission of the production systems. Requests to access the datasets should be directed to gustavos@iastate.edu.
